#  Genetic Association of Lipids and Lipid Drug Targets With Abdominal Aortic Aneurysm: A Meta-analysis

**DOI:** 10.1001/jamacardio.2017.4293

**Published:** 2017-11-29

**Authors:** Seamus C. Harrison, Michael V. Holmes, Stephen Burgess, Folkert W. Asselbergs, Gregory T. Jones, Annette F. Baas, F. N. van ’t Hof, Paul I. W. de Bakker, Jan D. Blankensteijn, Janet T. Powell, Athanasios Saratzis, Gert J. de Borst, Daniel I. Swerdlow, Yolanda van der Graaf, Andre M. van Rij, David J. Carey, James R. Elmore, Gerard Tromp, Helena Kuivaniemi, Robert D. Sayers, Nilesh J. Samani, Matthew J. Bown, Steve E. Humphries

**Affiliations:** 1 Cambridge Vascular Unit, Addenbrookes Hospital, Cambridge, England; 2 Cardiovascular Epidemiology Unit, University of Cambridge, Cambridge, England; 3 Clinical Trial Service Unit and Epidemiological Studies Unit, Nuffield Department of Population Health, University of Oxford, Oxford, England; 4 Medical Research Council Population Health Research Unit, Nuffield Department of Population Health, University of Oxford, Oxford, England; 5 National Institute for Health Research, Oxford Biomedical Research Centre, Oxford University Hospital, Oxford, England; 6 Medical Research Council Biostatistics Unit, University of Cambridge, Cambridge, England; 7 Department of Cardiology, Division of Heart and Lungs, University Medical Center Utrecht, Utrecht, the Netherlands; 8 Department of Epidemiology, Julius Center for Health Sciences and Primary Care, University Medical Center Utrecht, Utrecht, the Netherlands; 9 Department of Medical Genetics, Centre for Molecular Medicine, University Medical Center Utrecht, Utrecht, the Netherlands; 10 Farr Institute of Health Informatics Research and Institute of Health Informatics, University College London, London, England; 11 Department of Surgery, University of Otago, Dunedin, New Zealand; 12 Brain Center Rudolf Magnus, Department of Neurology and Neurosurgery, University Medical Center Utrecht, Utrecht, the Netherlands; 13 Department of Surgery, VU University Medical Center, Amsterdam, the Netherlands; 14 Vascular Surgery Research Group, Imperial College Charing Cross Hospital, London, England; 15 National Institute for Health Research Leicester Cardiovascular Biomedical Research Unit and Department of Cardiovascular Sciences, University of Leicester, Leicester, England; 16 Vascular Surgery, University Medical Center Utrecht, Utrecht, the Netherlands; 17 Institute of Cardiovascular Science, University College London, London, England; 18 Department of Medicine, Imperial College London, Hammersmith Hospital, London, England; 19 Sigfried and Janet Weis Center for Research, Geisinger Health System, Danville, Pennsylvania; 20 Department of Vascular and Endovascular Surgery, Geisinger Health System, Danville, Pennsylvania; 21 Division of Molecular Biology and Human Genetics, Department of Biomedical Sciences, Faculty of Medicine and Health Sciences, Stellenbosch University, Tygerberg, South Africa; 22 Department of Cardiovascular Genetics, Institute of Cardiovascular Science, University College London, London, England

## Abstract

**Importance:**

Risk factors for abdominal aortic aneurysm (AAA) are largely unknown, which has hampered the development of nonsurgical treatments to alter the natural history of disease.

**Objective:**

To investigate the association between lipid-associated single-nucleotide polymorphisms (SNPs) and AAA risk.

**Design, Setting, and Participants:**

Genetic risk scores, composed of lipid trait–associated SNPs, were constructed and tested for their association with AAA using conventional (inverse-variance weighted) mendelian randomization (MR) and data from international AAA genome-wide association studies. Sensitivity analyses to account for potential genetic pleiotropy included MR-Egger and weighted median MR, and multivariable MR method was used to test the independent association of lipids with AAA risk. The association between AAA and SNPs in loci that can act as proxies for drug targets was also assessed. Data collection took place between January 9, 2015, and January 4, 2016. Data analysis was conducted between January 4, 2015, and December 31, 2016.

**Exposures:**

Genetic elevation of low-density lipoprotein cholesterol (LDL-C), high-density lipoprotein cholesterol (HDL-C), and triglycerides (TG).

**Main Outcomes and Measures:**

The association between genetic risk scores of lipid-associated SNPs and AAA risk, as well as the association between SNPs in lipid drug targets (*HMGCR*, *CETP*, and *PCSK9*) and AAA risk.

**Results:**

Up to 4914 cases and 48 002 controls were included in our analysis. A 1-SD genetic elevation of LDL-C was associated with increased AAA risk (odds ratio [OR], 1.66; 95% CI, 1.41-1.96; *P* = 1.1 × 10^−9^). For HDL-C, a 1-SD increase was associated with reduced AAA risk (OR, 0.67; 95% CI, 0.55-0.82; *P* = 8.3 × 10^−5^), whereas a 1-SD increase in triglycerides was associated with increased AAA risk (OR, 1.69; 95% CI, 1.38-2.07; *P* = 5.2 × 10^−7^). In multivariable MR analysis and both MR-Egger and weighted median MR methods, the association of each lipid fraction with AAA risk remained largely unchanged. The LDL-C–reducing allele of rs12916 in *HMGCR* was associated with AAA risk (OR, 0.93; 95% CI, 0.89-0.98; *P* = .009). The HDL-C–raising allele of rs3764261 in *CETP* was associated with lower AAA risk (OR, 0.89; 95% CI, 0.85-0.94; *P* = 3.7 × 10^−7^). Finally, the LDL-C–lowering allele of rs11206510 in *PCSK9* was weakly associated with a lower AAA risk (OR, 0.94; 95% CI, 0.88-1.00; *P* = .04), but a second independent LDL-C–lowering variant in *PCSK9* (rs2479409) was not associated with AAA risk (OR, 0.97; 95% CI, 0.92-1.02; *P* = .28).

**Conclusions and Relevance:**

The MR analyses in this study lend support to the hypothesis that lipids play an important role in the etiology of AAA. Analyses of individual genetic variants used as proxies for drug targets support LDL-C lowering as a potential effective treatment strategy for preventing and managing AAA.

## Key Points

**Question:**

What is the association between genetically elevated lipid levels and the risk for abdominal aortic aneurysm?

**Findings:**

In this meta-analysis of up to 4914 cases and 48 002 controls in 5 genome-wide association studies, genetic elevation of low-density lipoprotein cholesterol and triglyceride levels were associated with an elevated risk of abdominal aortic aneurysm and high-density lipoprotein cholesterol level was associated with a lower risk of abdominal aortic aneurysm.

**Meaning:**

Patients with abdominal aortic aneurysm have a high burden of genetically determined dyslipidemia; targeting lipids in this high-risk group may improve longer-term outcomes.

## Teaser

This meta-analysis examines data sets from 5 international genome-wide association studies to determine the association between elevated lipids and the risk for abdominal aortic aneurysm.

## Introduction

Abdominal aortic aneurysm (AAA) is an important cardiovascular disease (CVD) resulting in approximately 4500 deaths from AAA rupture per year in the United States.^1^ Approximately 45 000 operations are carried out each year to prevent rupture, resulting in 1400 deaths.^1^ Screening for AAA reduces the burden of rupture,^2^ and therefore many countries now offer such screening to at-risk groups.^3,4^ The US Preventive Services Task Force recommends screening men aged 65 to 75 years with a history of smoking, and the American Heart Association guidelines suggest surgical repair is needed when the AAA reaches 5.5 cm in diameter.

Abdominal aortic aneurysm shares risk factors with occlusive atherosclerotic disease, but the magnitude and direction of this association is not always consistent. A growing body of evidence suggests considerable heterogeneity of risk factor associations among different forms of CVDs.^5,6,7^ For example, the risk of smoking for AAA is at least 2-fold greater than that for coronary heart disease (CHD),^7^ whereas type 2 diabetes appears to be protective for AAA but is a major risk factor for occlusive vascular disease.^6^ This example suggests that AAA may have some distinct causal pathways, and understanding these pathways is important for setting public health policies aimed at reducing the risk posed by AAA and its complications.

Genome-wide association studies (GWASs) of AAA have identified robust associations of loci that have previously been found for CHD (*9p21*),^8^ *DAB2IP* (Entrez Gene 153090),^9^ *LDLR* (Entrez Gene 3949),^10^ *SORT1* (Entrez Gene 6272),^11^ and *IL6R* (Entrez Gene 3570)^12^ as well as a number of variants that do not appear to be associated with other CVDs (*LRP1* [Entrez Gene 4035],^13^ *SMYD2* [Entrez Gene 56960], *ERG* [Entrez Gene 2078], *MMP9* [Entrez Gene 4318], and *LINC00540* [Entrez Gene 100506622]^14^). Again, these findings lend support to the hypothesis that AAA and CHD have overlapping pathophysiology, but the association with AAA and not with other CVDs suggests that discrete etiological pathways may well exist between these vascular diseases.

The role of low-density lipoprotein cholesterol (LDL-C) levels in CHD is well defined, and LDL-C lowering therapies are of clear benefit in reducing CHD risk.^15^ Genetic studies appear to support a causal role for hypertriglyceridemia in CHD,^16,17,18^ but genetic and clinical studies have cast doubt on the status of high-density lipoprotein cholesterol (HDL-C) as a causal factor in CHD.^16,18,19,20,21^ In AAA, meta-analyses of observational studies do show a consistent inverse association of HDL-C with AAA risk, but the association with LDL-C is less clear.^22,23^ It is important, however, to recognize that the studies included in these meta-analyses were small case-control studies, many of which did not adjust for statin use. There is a paucity of any data reporting an association between triglycerides (TG) and AAA risk or progression. From a clinical point of view, it is important to understand the role of lipids in AAA, especially considering the excess cardiovascular risks in patients with AAA^24^ and the recent publications showing low prevalence of lowering levels of LDL-C in patients with AAA.^25,26^ Previous genetic association studies have pointed to a potential role of lipids in AAA pathology,^10,11,27^ but this current study uses a larger panel of single-nucleotide polymorphisms (SNPs), a considerably larger sample, and more advanced methods.

Mendelian randomization (MR) is an approach that uses the unique properties of genotype to investigate causal associations.^28^ Specifically, genotype is randomly allocated at conception (owing to Mendel’s second law, a feature that is exploited to minimize confounding) and is not affected by reverse causation. Although MR has traditionally been used to explore causal associations between circulating biomarkers and disease phenotypes, it has an extension that uses genotype to validate drug targets. In this approach, variants in genes encoding potential drug targets are used as instruments to explore the utility of targeting this pathway in specific disease states.^29,30^ A major challenge in MR studies of complex traits such as lipid fractions is genetic pleiotropy, whereby SNPs influence circulating concentrations of multiple lipid fractions. This so-called pleiotropy may reflect an association of an SNP (or multiple SNPs in combination) with multiple discrete pathways that may have differing associations with AAA, leading to a potentially biased estimate from MR. Recent developments in the technique, such as multivariable MR,^16^ weighted median MR,^31^ and MR-Egger,^32^ have been used to address these issues, but pleiotropy still poses a challenge.

In this study, conventional inverse-variance weighted MR, multivariable MR, weighted median MR, and MR-Egger approaches were used to investigate the role of lipids in the etiology of AAA.

## Methods

From January 9, 2015, to December 21, 2016, we investigated the association of genetic risk scores (GRS) for lipid traits with AAA reported in up to 4914 cases and 48 002 controls across 5 international AAA GWASs^14^ that took place in the United Kingdom and Australia,^13,14^ New Zealand,^13,14^ the United States,^14^ the Netherlands, and Iceland.^9^ The GRS were composed of SNPs that are robustly associated with serum lipids in the Global Lipids Genetics Consortium meta-GWAS of circulating lipid levels.^33^ Data collection for this study took place between January 9, 2015, and January 4, 2016. Data analysis was conducted between January 4, 2015, and December 31, 2016.

### Study Populations

We used summary SNP-AAA association statistics from the 5 published GWASs of AAA. Detailed descriptions of these GWAS analyses are provided in the eAppendix in the Supplement and previous publications.^9,13,14^ We supplemented the study of single variants in genes encoding lipid drug targets with data derived from the Secondary Manifestations of Arterial Diseases (SMART) study. The Table includes the number of cases and controls in each study. Descriptions of study cohorts and demographic details are presented in the eAppendix in the Supplement and previous publications.^9,13,14^ In all studies, the case definition of AAA was an infrarenal aortic diameter of 3 cm or more by ultrasound or computed tomographic imaging or previous AAA rupture or repair. Details of the association tests and quality control used in each study are included in the eAppendix in the Supplement and a published meta-GWAS.^14^

**Table.** Summary of Abdominal Aortic Aneurysm Genome-Wide Association Studies

GWAS Data Set	Cases, No.	Controls, No.	Notes
Aneurysm Consortium (United Kingdom and Australia)^a^	1866	5435	WTCCC Common Control Group, nonscreened
Vascular Genetics Study (New Zealand)^a^	1005	996	Screened AAA-negative controls (<2.5 cm); 80% AAA >5 cm
GWAS (United States)^a^	724	1870	Cases identified in electronic health records, nonscreened
deCODE Genetics (Iceland)^a^	479	36 910	Nonscreened population
GWAS (the Netherlands)^a^	840	2791	Nonscreened population
SMART^b^	631	6342	AAA-negative controls with arterial disease^c^

Abbreviations: AAA, abdominal aortic aneurysm; GWAS, genome-wide association study; NA, not applicable; SMART, Secondary Manifestations of Arterial Diseases study; WTCCC, Wellcome Trust Case Control Consortium.

^a^ This cohort was used in the mendelian randomization of lipids (genetic risk score) analysis.

^b^ This cohort was used in the mendelian randomization of drug targets analysis.

^c^ Reflecting a single variant study only.

### Selection of SNPs

We identified SNPs associated with lipids in the Global Lipid Genetics Consortium^33^ using the SNP selection criteria by Do et al.^16^ Briefly, SNPs in association with at least 1 of the 3 lipid traits (LDL-C, HDL-C, or TG concentrations) at a genome-wide significance level (*P* < 5 × 10^−8^) were selected. In Do et al^16^ at loci with multiple associated SNPs, single SNPs with the strongest effect estimates were selected, and more than 1 SNP was selected only if there was evidence of minimal linkage disequilibrium (*r*^2^ < 0.05). Data were available for the 180 of 185 SNPs (eTable 1 in the Supplement) described in Do et al.^16^

### Data Analysis

We first harmonized SNPs across the data sets (Global Lipids Genetics Consortium and Aneurysm Consortium) by merging SNPs on the reference SNP cluster identification or rs number. Then, we ensured that effect alleles were denoted to be the same in both data sets and double-checked the information by investigating effect-allele frequencies. We oriented all variants to ensure that the effect allele was positively associated with each lipid trait (eg, in the MR of LDL-C, all β coefficients for LDL-C were >0). This orientation resulted in a data set in which each SNP was a unique row and there were separate columns for β and SEs for each lipid trait and the log odds ratio (OR) and corresponding SE for AAA (eTable 1 in the Supplement).

### Conventional MR

We conducted a conventional 2-sample MR analysis to determine the association between a 1-SD genetically elevated lipid concentration and AAA risk. For this analysis, we used the inverse-variance weighted MR method in which the SNP association estimates for the outcome (β for AAA) are regressed on the SNP association estimates for each lipid (β for LDL-C, β for HDL-C, and β for TG) individually in turn. The regression was weighted by the inverse variances of the estimated associations of the SNPs with the outcome and then was forced to pass through the origin.

### Multivariable MR

To gauge some insight into potential “independent” associations of the lipids with AAA risk, we used the multivariable MR method. In this approach, a single regression model with outcome variable (β for AAA) was fitted for the predictor variables (β for LDL-C, β for HDL-C, and β for TG). The model was implemented, as described previously,^34^ as a multilinear regression of SNP association estimates weighted by the inverse variances of the estimated associations of SNPs with the outcome and forced to pass through the origin.

### MR-Egger

We used the MR-Egger^32^ method that tests for the presence of, and provides an MR estimate that is adjusted for, unmeasured net pleiotropy. The method involves conducting an unconstrained linear regression of the SNP association estimates for the outcome on the SNP association estimates for the exposure weighted by the inverse variance of the estimated association of SNP with outcome. In MR-Egger, any net pleiotropy manifests in the intercept. Under the assumption that pleiotropic associations are independent of the associations of the SNPs with the exposure, the regression slope coefficient should represent an unbiased MR association estimate.

### Weighted Median MR

As a further sensitivity analysis, we performed the weighted median MR method.^31^ Whereas the conventional inverse-variance weighted method calculates a weighted mean of the SNP-specific causal association estimates, the weighted median method calculates a weighted version of the median of the SNP-specific causal association estimates. Because the median of a distribution is not affected by extreme values, the weighted median method is less sensitive to individual pleiotropic SNPs. The weighted median estimate is unbiased in large samples if at least 50% of the weights from SNPs are valid (eg, not pleiotropic).

### SNPs in Drug Target Analysis

To our knowledge, there have been no large-scale randomized trials of lipid-lowering treatments in patients with AAA, and observational studies have often been small and retrospective and yielded heterogeneous results. We examined the association of rs12916 in *HMGCR* (a genetic proxy for statins; Entrez Gene 3156), rs3764261 in *CETP* (a proxy for CETP inhibitors; Entrez Gene 1071), as well as rs2479409 and rs11206510 in *PCSK9* (a proxy for PCSK9 inhibitors; Entrez Gene 255738) with AAA to identify the potential utility of pharmacological modification of these drug targets in AAA.

### Statistical Calculations

The MR analyses for blood lipids were performed using the “MendelianRandomization” command in R, version 3.3.3 (R Foundation for Statistical Computing),^35^ and 2-tailed *P* values were derived from instrumental variable estimators. Given that there was only one outcome under investigation (AAA) and the lipids traits were correlated with one another, we used 2-tailed *P* < .05 to denote evidence against the null hypothesis (ie, *P* < .05 provided evidence in favor of an association between the exposure and outcome).

## Results

The numbers of cases and controls for each of the 5 AAA GWASs are shown in the Table. Up to 4914 cases and 48 002 controls were included in our analysis. The complete list of SNPs analyzed in this study, together with information on the association statistics for AAA, and for LDL-C, HDL-C, and TG levels, is included in eTable 1 in the Supplement.

### Conventional Inverse-Variance Weighted MR: Association of GRS With AAA

Summary statistics for 180 lipid-associated SNPs were available for analysis. As previously reported,^11,14^ the LDL-C–lowering alleles of rs6511720 in *LDLR* (OR per allele, 0.75; 95% CI, 0.67-0.83; *P* = 5.2 × 10^−12^) and rs646776 in *SORT1* (OR per allele, 0.88; 95% CI, 0.82-0.94; *P* = 3.9 × 10^−8^) were strongly associated with AAA. No other SNP from the 180 lipid-associated SNPs was individually associated with AAA at conventional levels of genome-wide significance (*P* < 5.0 × 10^−8^). Twenty-five of 180 SNPs (13.8%) were nominally associated with AAA (*P* < .05; eTable 2 in the Supplement) with 9 such associations (95% CI, 4-15) being expected by chance alone.

**Figure 1.**
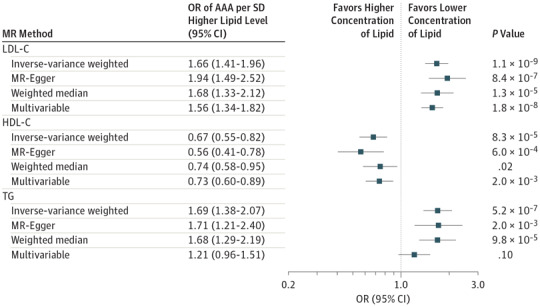
Association of Lipid Genetic Risk Scores With Abdominal Aortic Aneurysm (AAA) Risk The 4 different mendelian randomization (MR) methods used to determine this association were conventional inverse weighted MR, MR-Egger, weighted median MR, and multivariable MR. LDL-C indicates low-density lipoprotein cholesterol; HDL-C, high-density lipoprotein cholesterol; OR, odds ratio; and TG, triglycerides (TG).

We conducted conventional inverse-variance weighted MR analyses using GRS for LDL-C (75 SNPs), HDL-C (84 SNPs), and TG levels (50 SNPs) to assess the associations with AAA (Figure 1). The LDL-GRS was strongly associated with AAA risk (OR per SD higher level for LDL-C, 1.66; 95% CI, 1.41-1.96; *P* = 1.1 × 10^−9^). A 1-SD higher HDL-C level instrumented through the HDL-C GRS was associated with a reduced AAA risk (OR, 0.67; 95% CI, 0.55-0.82; *P* = 8.3 × 10^−5^). In addition, the TG-GRS was associated with higher AAA risk (OR per 1-SD higher TG level, 1.69; 95% CI, 1.38-2.07; *P* = 5.2 × 10^−7^).

### Multivariable MR, MR-Egger, and Weighted Median MR Approaches

It is possible to remove SNPs with pleiotropic associations from the GRS, but this removal diminishes the strength of the instrumental variable^36^ and can introduce bias.^37^ Therefore, we adopted the multivariable MR method described by Do et al^16^ and modified by Burgess and Thompson^34^ to gain insight into the potential independent associations of these lipid GRS with AAA risk. To account for any net unbalanced pleiotropy, we used the MR-Egger method. To reduce the influence of outlying (possibly pleiotropic) variants on the analysis, we used the weighted median MR method. None of these sensitivity MR analyses resulted in a material change to either the magnitude or significance of the estimates (Figure 1). The point estimates for concentrations of LDL-C and HDL-C remained largely unaltered, whereas for TG the point estimate diminished for the multivariable MR method; however, on the MR-Egger and weighted median MR methods, TG level remained convincingly associated with AAA.

### Association of SNPs With Lipid Drug Targets

We selected rs12916 in *HMGCR*, rs3764261 in *CETP*, as well as rs2479409 and rs11206510 in *PCSK9* as there are licensed drugs that target pathways associated with these genes.

**Figure 2.**
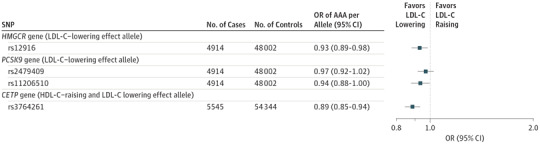
Association of Single-Nucleotide Polymorphisms (SNPs) in Genes Encoding Drug Targets With Abdominal Aortic Aneurysm (AAA) Risk SNPs were proxies for lipid drug targets. Analysis of *CETP* gene included additional cases and controls from the Secondary Manifestations of Arterial Diseases (SMART) study. LDL-C indicates low-density lipoprotein cholesterol; HDL-C, high-density lipoprotein cholesterol; and OR, odds ratio.

The LDL-C–lowering allele of rs12916 (to proxy statin use) was associated with a lower AAA risk in meta-analysis (OR per LDL-C–lowering allele, 0.93; 95% CI, 0.89-0.98; *P* = .009) (Figure 2).

The PCSK9 inhibitors are a novel class of drugs used to target LDL-C. To date, in CHD, genetic and clinical studies have had concordant results.^33,38^ We examined 2 independent SNPs in *PCSK9* (rs2479409 and rs11206510; linkage disequilibrium *r^2^* = 0.07) that were used as proxies for PCSK9 inhibition in a large-scale MR analysis^39^ and have strong, independent associations with both LDL-C levels and CHD. The LDL-C–lowering allele of rs2479409 was not associated with AAA risk (OR, 0.97; 95% CI, 0.92-1.02; *P* = .28). The LDL-C–lowering allele of rs11206510 in *PCSK9* was weakly associated with AAA risk (OR, 0.94; 95% CI, 0.88-1.00; *P* = .04) (Figure 2).

We used rs3764261 as a proxy for CETP inhibition. Although the allele increases HDL-C levels, it is also associated with lower circulating concentrations of TG and LDL-C; thus, rs3764261 cannot be considered as an instrument for HDL-C in isolation but can be used to gauge insight into the potential associations with CETP inhibition.^30^ This HDL-raising *CETP* SNP was associated with lower AAA risk (OR per HDL-C–raising allele, 0.89; 95% CI, 0.85-0.94; *P* = 3.7 × 10^−7^).

## Discussion

Understanding the relevance of lipid fractions in the development of AAA has important implications from both etiological and translational standpoints. In this study, we used MR to provide robust evidence that the major lipid fractions—LDL-C, HDL-C, and TG—are likely to play important roles in the etiology of AAA. A similar genetic approach has been used previously,^27^ but this present study has expanded on this technique by including many more individuals and more SNPs and by using more recent developments in MR, which collectively increase statistical power and strengthen the validity of the association estimates reported here.

Disentangling the roles of correlated biomarkers in disease etiology continues to be an analytical challenge; to this end, we used recently developed techniques for the multivariable MR method.^16^ Interestingly, there appear to be independent associations between genetically instrumented levels of LDL-C, HDL-C, and TG and AAA risk. This finding is in contrast to findings in studies of CHD in which a similar approach found weaker associations between HDL-C genetic variants and CHD (after shared pathways with LDL-C and TG and pleiotropy had been taken into account^16,18,19,36^) or aortic stenosis in which only LDL-C appeared to play a causal role.^40^ This finding highlights the complexity of lipid pathways across the diverse biology of CVD and suggests that results from studies focused solely on CHD (which can be defined variably) cannot always be extrapolated to other vascular diseases such as AAA.

Although it has been possible to investigate for pleiotropic associations of genetic variants used collectively in the lipid GRS employed in the MR analyses we conducted, it is not so straightforward as to disentangle the phenotypic overlap whereby many patients with AAA also harbor atherosclerotic disease in other vascular beds. Therefore, it is tempting to suggest a causal role for lipids specifically in AAA pathogenesis, but these genetic analyses do not provide definitive evidence. The data do suggest, however, that the burden of genetically influenced dyslipidemia in patients with AAA is considerable, and by extrapolation, these MR analyses lend support to the lipids playing an important role in AAA etiology and thus targeting lipids through pharmacological modification in patients with small AAAs may well be justified. This point is particularly pertinent given the recent reports of low prevalence of control of LDL-C concentrations in patients with AAA in both the United States and the United Kingdom.^25,26^ In addition, this group of patients should be considered in trials evaluating novel treatments of lipid-lowering medications, such as CETP or PCSK9 inhibitors.

The use of genetic data to inform drug trials and/or drug repurposing represents an important translational facet of data derived by large genome-wide consortia.^41,42^ In addition to the GRS for LDL-C, HDL-C, and TG, we looked at 4 loci that serve as proxies for cardiovascular drug targets that have not been subjected to clinical trials in patients with AAA. Both the LDL-C GRS and a genetic proxy for statin therapy (SNPs in *HMGCR*) were associated with AAA. Previous investigations on the associations of concentrations of LDL-C with AAA have used cross-sectional data sets with varying findings, and results have been hampered by concurrent LDL-C–lowering therapies.^43^ Indeed, there has been a suggestion that statin use may increase AAA risk.^44^ The collective results from this study suggest that LDL-C plays an important role in the etiology of AAA, which may explain the excess burden of CVD in patients with AAA.^24^ These data also support a view that patients found by screening to have AAA should be prescribed statins to reduce their CVD risk, although whether this will affect the progression of AAA cannot be answered in this study.

A recent phase 3 clinical trial showed that PCSK9 inhibitors have beneficial effects on CVD outcomes.^38^ Although the association we found between *PCSK9* variants and AAA was weak, if PSCK9 inhibitors do prove to be a safe and cost-effective means of lowering LDL-C levels, then consideration should be given to evaluating these drugs in patients with AAA.

As noted, a genetically instrumented higher HDL-C level was identified to be associated with a reduction in AAA risk. Variants in *CETP* have a range of results similar to pharmacological inhibition of CETP,^30^ including lowering of LDL-C and raising of HDL-C levels. A trial of CETP inhibition showed modest benefit in patients following myocardial infarction,^45^ and there are data to support its beneficial effects on vascular remodeling^46^ that could have relevance in AAA management. Evaluation of CETP inhibition in patients with AAA may therefore be warranted. Although we cannot specifically determine whether the association between *CETP* polymorphisms and AAA is via HDL-C, LDL-C, or TG (or indeed all, as suggested by our GRS of lipid traits), we believe our results suggest that CETP inhibition could play a role in the management of AAA.

The findings regarding TG variants also have potential clinical implications for the development of novel treatments aimed at TG levels. They suggest that patients with AAA may benefit from lowering TG levels. As novel therapies such as APOC3 inhibitors progress from phase 2 studies to larger-scale phase 3 studies of CVD prevention, then patients with AAA could be an important CVD subphenotype in whom treatment should be evaluated.

Our study used MR, a genetic approach that has important assumptions. The SNPs used in the genetic instruments for each lipid trait were identified from recent GWASs that placed stringent thresholds on SNP discovery. As such, the genetic instruments are very unlikely to suffer from weak instrument bias; in any case, because the MR analyses used nonoverlapping data sets, such bias would tend to dilute the estimates derived from MR analyses.^47^ In addition, we made the assumption that the genetic instruments are not influenced by confounding and that they only associate with AAA through the exposure of interest (ie, the genetic instruments are not affected by unbalanced horizontal pleiotropy, as pictorially illustrated in Figure 1 of White et al^18^ and expanded in Holmes et al^37^). These assumptions cannot be tested with complete certainty. However, causal estimates obtained from a range of sensitivity analyses, each making different and weaker assumptions, all gave similar results. Nonetheless, residual pleiotropy could still influence our findings.

### Limitations

The limitations of this study should be considered. First, we did not have data sets to evaluate AAA progression. Second, owing to limited availability of covariate data, we were unable to examine the influence of concurrent lipid-lowering therapy on the estimates derived from the GRS for blood lipid traits and AAA risk. Third, our analyses used summary-level data as described elsewhere.^16,48^ Use of summary-level data can hamper more refined analyses (eg, subgroup analyses by sex or age), but one of its main strengths is it facilitates 2-sample MR analyses of the type reported here. This greatly strengthens the power of the study, which enables the conduct of sensitivity analyses (such as MR-Egger and weighted median MR methods) and the investigation of certain instrumental variable assumptions such as the absence of genetic pleiotropy. Finally, although we attempted to control for pleiotropy in the analyses, we believe pleiotropy still represents a major challenge to deciphering the roles of specific lipid-based pathways.

## Conclusions

Using contemporary MR approaches, we found data that lend support to the hypothesis that major lipid fractions are involved in the etiology of AAA. Consideration should be given to measures aimed at targeting lipids to reduce risk of AAA, using established and emerging therapies.

## Notes

Supplement. **eAppendix.** Methods **eTable 1.** The Effect Size for 180 SNPs on AAA, CHD (from the Cardiogram Dataset) and Lipid Fractions (from the Global Lipid Genetics Consortium GWAS), Expressed as Beta Coefficients and Standard Errors (e.g., AAA_beta and AAA_se) **eTable 2.** Association of Lipid GRS with CHD in the CARDIoGRAMplusC4D Dataset Using Inverse Weighted MR and MR-Egger
eReferences
