# Supplementary material for: Genetic Association of Lipids and Lipid Drug Targets With Abdominal Aortic Aneurysm: A Meta-analysis
Source: JAMA Cardiol. 2017 Nov 29;3(1):26–33. doi: 10.1001/jamacardio.2017.4293 (PMC5833524; doi:10.1001/jamacardio.2017.4293)
Supplement: Supplement. — eAppendix. Methods eTable 1. The Effect Size for 180 SNPs on AAA, CHD (from the Cardiogram Dataset) and Lipid Fractions (from the Global Lipid Genetics Consortium GWAS), Expressed as Beta Coefficients and Standard Errors (e.g., AAA_beta and AAA_se) eTable 2. Association of Lipid GRS with CHD in the CARDIoGRAMplusC4D Dataset Using Inverse Weighted MR and MR-Egger eReferences [file jamacardiol-e174293-s001.pdf]

Supplementary Online Content

Harrison SC, Holmes MV, Burgess S, et al. Genetic association of lipids and lipid drug targets with abdominal aortic aneurysm: a meta-analysis. *JAMA Cardiol.* Published online November 29, 2017. 10.1001/jamacardio.2017.4293

eAppendix. Methods

eTable 1. The Effect Size for 180 SNPs on AAA, CHD (from the Cardiogram Dataset) and Lipid Fractions (from the Global Lipid Genetics Consortium GWAS), Expressed as Beta Coefficients and Standard Errors (e.g. AAA_beta and AAA_se)

eTable 2. Association of Lipid GRS with CHD in the CARDIoGRAMplusC4D Dataset Using Inverse Weighted MR and MR-Egger

eReferences

This supplementary material has been provided by the authors to give readers additional information about their work.

© 2017 American Medical Association. All rights reserved.

Table of Contents

eAppendix. Methods
Cohorts
The Aneurysm Consortium
New Zealand (NZ) Vascular Genetics Study AAA GWAS dataset
US (PA) GWAS dataset
Iceland, deCODE Genetics AAA GWAS dataset
The Netherlands AAA GWAS dataset
SMART study
Association of Lipid GRS with CHD

Supplementary tables

eTable 1. The effect size for 180 SNPs on AAA, CHD (from the Cardiogram dataset) and Lipid Fractions (from the Global Lipid Genetics Consortium GWAS), expressed as beta coefficients and standard errors (e.g. AAA_beta and AAA_se). This table is provided as a separate Excel file.

eTable 2. Association of lipid GRS with CHD in the CARDIoGRAMplusC4D dataset using inverse weighted MR and MR-Egger.

eReferences

© 2017 American Medical Association. All rights reserved.

eAppendix. Methods

Cohorts

The GWAS cohorts used in this MS are described in detail in the recent meta-analysis of AAA GWAS study(1).

The Aneurysm Consortium

Aneurysm Consortium (AC) AAA GWAS dataset: The AC recruited cases of AAA from centers across the United Kingdom and Western Australia. Cases were defined as patients with an infra-renal aortic diameter ≥ 30 mm proven on ultrasound or computerized tomography (CT) scan. Controls were taken from the Wellcome Trust Case Control Consortium 2 (WTCCC2) common control group(2) and were, therefore, unscreened for AAA.

Data from 1,866 cases with AAA and 5,435 unscreened controls from the WTCCC2 study consisting of samples from the 1958 British Birth Cohort and from the UK National Blood Service. DNA samples were processed at the Wellcome Trust Sanger Institute (WTSI). Genomic DNA was quantified by PicoGreen assay, and quality control (QC) assured by both agarose gel electrophoresis and Sequenom iPLEX genotyping of 29 SNPs and 4 sex-specific markers. Genotyping for the discovery study was performed using Illumina 1.2M (controls) or 670K (AAA) BeadChips.

As part of the original AC GWAS individual sample QC had been performed as follows. QC was first performed by exclusion of SNPs with call rates < 0.98 and those that demonstrated significant deviation from Hardy-Weinberg equilibrium in the control group ($P<5x10^{-4}$). Duplicate samples and those that failed genotyping (sample call rates <0.98) were also excluded from further analysis. Genotyping cluster plots for all SNPs with $P< 1x10^{-4}$ were visually inspected to exclude from further analysis positive associations generated by erroneous genotyping or calling. Checks for population stratification were performed by PLINK identical by state clustering and extreme outliers were removed from the analysis.

Imputation was performed using IMPUTE 2.2 run on the BCISNPmax database platform (version 3.5, BCI Platforms, Espoo, Finland). The reference haplotypes were based on the 1000 Genomes June 2011 release. Imputed calls were filtered by quality score (excluding those <0.9) to restrict to higher quality imputed SNPs.

Following imputation further QC filtering was performed, excluding SNPs with call rates <0.98 and those that demonstrated significant deviation from Hardy-Weinberg equilibrium in the control group ($P<5x10^{-4}$). Duplicate samples and those that failed genotyping (sample call rate <0.98) were also excluded from further analysis. Association testing was carried out in PLINK.

New Zealand (NZ) Vascular Genetics Study AAA GWAS dataset

The Vascular Research Consortium of New Zealand recruited New Zealand men and women with a proven history of AAA (infra-renal aortic diameter ≥ 30 mm proven on ultrasound or CT scan). Approximately 80% had undergone surgical AAA repair (typically AAA's > 50-55 mm in diameter). The vast majority of cases (>97%) were of Anglo-European ancestry. The control group underwent an abdominal ultrasound scan

© 2017 American Medical Association. All rights reserved.

to exclude (>25 mm) concurrent AAA and Anglo-European ancestry was required for inclusion. Controls were also screened for peripheral artery disease (PAD; using ankle brachial index), carotid artery disease (ultrasound) and other cardiovascular risk factors.

Two separate GWAS were performed using New Zealand samples. NZ GWAS 1 consisted of 608 AAA patients (474 male) and 612 elderly controls (450 male), genotyped using the Affymetrix SNP 6 GeneChip array. All samples had call rates >0.95 (mean 0.992). NZ GWAS 2 consisted of 397 AAA patients (332 male) and 384 elderly controls (308 male), genotyped using the Illumina Infinium Omni2.5 BeadChip array. All samples had call rates >0.95 (mean 0.990). All NZ genomic DNA samples exceeded manufacturers quality and quantity requirements having undergone pre-assessment by Nanophotometer (Implen GmbH, München, Germany) and agarose gel electrophoresis.

Imputation was conducted separately on NZ GWAS 1 and 2 data sets using the same methods as used for the AC datasets. IMPUTE 2.2 was run on the BCISNPmax database platform (version 3.5, BCI Platforms, Espoo, Finland). The reference haplotypes were based on the 1000 Genomes June 2011 release. Imputed calls were filtered by quality score (excluding those <0.9) to restrict to higher quality imputed SNPs. The genomic inflation factors ([illegible]) were 1.07 and 1.05 respectively (MAF >0.05).

Both NZ GWAS 1 and 2 data sets underwent QC filtering, excluding SNPs with call rates < 0.98 and those that demonstrated significant deviation from Hardy-Weinberg equilibrium in the control group ($P<5x10^{-4}$). Duplicate samples and those that failed genotyping (sample call rate <0.98) were also excluded from further analysis.

US (PA) GWAS dataset

AAA patients were enrolled through the Department of Vascular Surgery at Geisinger Medical Center, Danville, Pennsylvania, USA as previously reported. To identify cases and controls from the electronic medical records, an ePhenotyping algorithm was developed(3). Briefly, Structured Query Language (SQL) was used to script the algorithm utilizing "Current Procedural Terminology" (CPT) and "International Classification of Diseases" (ICD-9) codes as well as demographic and encounter data to classify individuals as case, control, or excluded. AAA cases were defined as having an AAA repair procedure (case Type 1), or at least one appropriate specialty encounter (vascular clinic) with a ruptured AAA (case Type 2), or at least two specialty encounters with an unruptured AAA (case Type 3). Controls were neither cases nor those excluded, had an encounter within the past 5 years, and had never been assigned an ICD9 code of 441.*, where * is a 1 or 2 digit code. Individuals were excluded if 1) they had a thoracic aortic aneurysm or a rare heritable disease with aortic manifestation; 2) they were younger than 40 or older than 89 years, 3) they had a single encounter with a code without mention of rupture (441.4), or 4) they had not had an encounter within the past 5 years. Rare heritable diseases were excluded because the goal of the current study was to identify non-syndromic AAA. Controls under 40 years might yet manifest an AAA, while cases under 40 years of age and without rare syndromic forms of aortic aneurysms are likely due to trauma. The AAA algorithm can be downloaded from www.PheKb.org. The algorithm was validated on a subset of individuals by manual chart review, and implemented at eMERGE network sites. The algorithm was implemented as a workflow in the Konstanz Information Miner (KNIME) (http://www.knime.org/).

© 2017 American Medical Association. All rights reserved.

AAA cases had infrarenal aortic diameter ≥30 mm as revealed by abdominal imaging. Approximately 20% of individuals with AAA had a family history of AAA. A control group was obtained through the Geisinger MyCode® Project, a cohort of Geisinger Clinic patients recruited for genomic studies. The MyCode® controls were matched for age distribution and sex to the Geisinger Vascular Clinic AAA cases. Based on electronic medical records, controls had no ICD-9 codes for AAA in their records, but they were not screened by ultrasonography for AAA. Both cases and controls from the Geisinger Clinic were of European descent.

The GWAS data used for this study was a subset of a larger cohort comprising 3,264 samples from 3,149 individuals with three phenotypes: 922 putative AAA cases, 981 obesity cases and 1,246 controls. Samples were genotyped on the Illumina HumanOmniExpress-12v1.0 genotyping platform at the University of Pittsburgh Genomics and Proteomics Core Laboratories. Genotypes were called using the Illumina GenomeStudio v2010.3 software. QC consisted of a number of steps: identification of cross-contamination and removal of specimens, call rate of samples (>0.98 SNPs called), sex consistency between annotated sex and genotyped sex, SNP discordance between replicate sample pairs, SNP call rate (>0.95 calls in all specimens), SNP minor allele frequency (>0.01), SNP Hardy-Weinberg equilibrium ($P > 1x\ 10^{-4}$), and selection of replicates to retain based on sex-specific Mahalanobis distance (<4.1) and Illumina P10.GC (>0.71). Cross-contamination of samples was detected by excess heterozygosity and excess relatedness (related to more than half of other samples at Pi-hat>0.0625); four samples were removed prior to other QC steps. After the QC steps above, related individuals (pairwise Pi-hat>0.15) were removed, retaining the individual and specimen with the highest call rate. A second round of QC applied using the above SNP and sample criteria to ensure consistency after removal of SNPs and individuals. In addition, the SNP criteria were analyzed per chromosome to ensure that there were no systematic differences (no differences detected). Lastly, principle component analysis (PCA) was used to determine if there were any batch effects during genotyping (no evidence for batch effects). Of the 3,264 samples, 153 were removed for one or more of the QC reasons above. Of the 731,306 SNPs, 95,369 were removed; 2,012 were discordant, 13,107 had a low call rate, 78,086 had a MAF<0.01, and 14,056 had a HWE $P<1x\ 10^{-4}$ (9,047 SNPs were removed for more than one reason).

The final meta-analysis cohort comprised only those individuals who were identified as definitive AAA cases or controls using the rigorous ePhenotyping algorithm described above.

Imputation was performed as previously described(4). Briefly: SNPs were re-mapped to the Genome Reference Consortium Human build 37 (GRCh37) and the program liftOver run to ensure mapping consistency. Subsequently all SNPs were mapped from the Illumina TOP notation to the plus (+) strand. Strand was checked using SHAPEIT2 (version r2.644)(5). Next the data were phased using SHAPEIT2. Imputation was performed using IMPUTE2 (version 2.3.0)(6). Chromosomes were divided into 6 MB segments with 250 kbp overlap between segments. A total of 5,719,283 SNPs with an info score of >=0.9 were used for analysis.

Association analysis without adjustment was performed using PLINK (v1.09)(7) and the imputed SNPs. The eMERGE Network Imputed GWAS for 41 Phenotypes (the dbGaP eMERGE Phase 1 and 2 Merged data Submission) accession number is: phs000888.v1.p1 which includes these AAA data.

© 2017 American Medical Association. All rights reserved.

Iceland, deCODE Genetics AAA GWAS dataset

Icelandic individuals with AAA (defined as infra-renal aortic diameter ≥ 30 mm) were recruited from a registry of individuals who were admitted at Landspitali University Hospital, in Reykjavik, Iceland, 1980 – 2006. AAA patients were either followed up or treated by intervention for emergency repair of symptomatic or ruptured AAA or for an elective repair by surgery or endovascular intervention. In total, whole genome data from subjects with AAA, enrolled as part of the cardiovascular disease (CVD) genetics program at deCODE, were included in the metaGWAS. The Icelandic controls used were selected from individuals who have participated in various GWA studies and who were recruited as part of genetic programs at deCODE. Individuals with known CVD were excluded as controls(8) but controls were unscreened for AAA.

The Icelandic case and control samples were assayed with the Illumina HumanHap300, HumanHapCNV370 or HumanHap610 bead chips (Illumina, San Diego, CA, USA). Only SNPs present on all chips were included in the analysis and SNPs were excluded if they had (a) call rates <95% in cases or controls, (b) MAF<0.01 in the controls, or (c) showed significant deviation from Hardy-Weinberg equilibrium in the controls ($P<1x10^{-4}$). These criteria were applied separately to genotype data from each of the chip types used and SNPs that showed significant deviation ($P<1x10^{-4}$ in an ANOVA test) in frequency between the chips were excluded from the analysis. Any samples with a call rate <98% were excluded from the analysis. The final analysis included 293,677 SNPs present on all three chips.

For case-control association analysis, we used a standard likelihood ratio statistic, implemented in the NEMO software(9)(9) to calculate two-sided P values and ORs for each individual allele, assuming a multiplicative model for risk(10).

The Netherlands AAA GWAS dataset

The AAA sample set from Utrecht was recruited in 2007-2009 from 8 centres in The Netherlands, mainly when individuals visited their vascular surgeon in the clinic or, in rare cases, during hospital admission for elective or emergency AAA surgery. An AAA was defined as an infrarenal aorta ≥ 30 mm. The sample set comprised 89.9% males, with a mean AAA diameter of 58.4 mm, 61.7% had been operated on, of which 8.1 % were after rupture. The Dutch controls used in the AAA GWAS were recruited as part of the Nijmegen Biomedical Study and the Nijmegen Bladder Cancer Study (see http://dceg.cancer.gov/icbc/membership.html).

Genotyping was performed on Illumina HumanHap610 chips. As controls, we included 2791 Dutch subjects who were recruited as part of the Nijmegen Biomedical Study (n=1,832) and the Nijmegen Bladder Cancer Study (n=1,278) These controls were genotyped on Illumina CNV370 Duo BeadChips.

We performed QC using PLINK version 1.07. After removal of SNPs with A/T or C/G alleles and SNPs that were not called in any individual, we performed sample QC and SNP QC.

Sample QC was performed after merging cases and controls, using a subset of common, high-quality SNPs [as defined by SNPs without deviation from Hardy-Weinberg equilibrium (P>0.001), with high MAF (>0.2) and with low rate of missing genotypes (<0.01)]. Linkage disequilibrium (LD) pruning (r^2>0.5) was performed. Subjects were removed based on the following three criteria: missing genotypes (subjects with call

© 2017 American Medical Association. All rights reserved.

rates<0.95 were removed), heterozygosity (subjects were excluded if the inbreeding coefficient deviated more than 3 standard deviations from the mean) and cryptic relatedness (by calculating identity-by-descent (IBD) for each pair of individuals). In each pair with an IBD proportion of >20%, a subject was excluded, if it exhibited distant relatedness with more than one individual. For case-control pairs, we removed the control subject. In the case-case or control-control pairs, the subject with the lowest call rate was excluded.

Using these common, high-quality SNPs, we performed PCA using EIGENSTRAT on the remaining study subjects and HapMap-CEU subjects. We excluded SNPs from three regions with known long-distance LD: the major histocompatibility (MHC) region (chr6: 25.8-36 Mbp), the chromosome 8 inversion (chr8: 6-16 Mbp) and a chromosome 17 region (chr17: 40-45 Mbp). We created PC plots with the first four PCs, using R version 2.11. Based on visual inspection of these plots, we excluded subjects that appeared to be outliers with respect to the CEU or the study population. After outlier removal, we recomputed PCs for them to be included as covariates in the logistic regression models.

After sample QC, we excluded SNPs with more than 2% missing genotypes, MAF<0.01, missing genotype rate higher than MAF, and HWE deviation (P<0.001). Because cases and controls had been genotyped separately, we performed these QC steps in each study cohort separately and again after merging cases and controls. We also removed SNPs with a differential degree of missing genotypes between cases and controls ($P<1x10^{-5}$; chi-squared test).

We performed genotype imputation using the pre-phasing/imputation stepwise approach implemented in IMPUTE2 and SHAPEIT (chunk size of 3 Mb and default parameters)(5,11). The imputation reference set consisted of 2,184 phased haplotypes from the full 1000 Genomes Project data set (February 2012; 40,318,253 variants). All genomic locations are given in NCBI Build 37/UCSC hg19 coordinates. After imputation, SNPs with an imputation accuracy score <0.6 or MAF <0.005 were excluded.

Association testing was carried out in PLINK(7) using imputed SNP dosages. We included as covariates the first four PCs. We calculated genomic inflation factors (λ_{GC}), defined as the ratio of the median of the empirically observed distribution of the test statistic to the expected median(12)

SMART study

The Secondary Manifestations of ARTerial disease (SMART) study is a prospective outpatient cohort study among patients aged 18-74 years newly referred to the University Medical Center Utrecht, The Netherlands, because of atherosclerotic vascular disease or for treatment of atherosclerotic risk factors. The objective of SMART is to determine the prevalence of concomitant asymptomatic arterial disease and risk factors in patients presenting with a manifestation of arterial disease or risk factor, and to study the incidence of future cardiovascular events and their predictors in these high-risk patients. Details of the protocol have been described elsewhere(13). DNA for wet-lab genotyping to replicate discovery results of the current study was available in a total of 8,361 SMART participants. There are 631 incident and prevalent AAA cases (defined as an infra-renal aortic diameter ≥3 cm) and 6,342 controls included in the analyses. There were 222 prevalent AAA end-points seen during follow. AAA endpoints included death, open surgical repair and/or endovascular stenting.

© 2017 American Medical Association. All rights reserved.

Association of Lipid GRS with CHD

As a positive control for our analysis we examined the association of lipid GRSs with CHD in the CARDIoGRAMplusC4D dataset(14) using the same methods as in the main manuscript. These findings are presented in eTable 2.

© 2017 American Medical Association. All rights reserved.

eTable 1. The Effect Size for 180 SNPs on AAA, CHD (from the Cardiogram Dataset) and Lipid Fractions (from the Global Lipid Genetics Consortium GWAS), Expressed as Beta Coefficients and Standard Errors (e.g. AAA_beta and AAA_se)

This table is provided as a separate Excel file.

rsID	Allele 1	Allele 2	AAA_beta	AAA_se	AAA P-Value	LDL_beta	HDL_beta	TG_beta	LDL_se	HDL_se	TG_se
rs6511720	t	g	-0.2848	0.0413	5.20E-12	-0.2209	0.0249	-0.0084	0.0061	0.0057	0.0056
rs646776	t	c	0.1644	0.0299	3.90E-08	0.1602	-0.034	0.0034	0.0044	0.0041	0.004
rs515135	t	c	-0.1218	0.0332	0.000241	-0.1394	0.011	-0.0192	0.0048	0.0044	0.0044
rs894210	a	g	-0.089	0.0247	0.00032	-0.0071	0.0689	-0.0671	0.0037	0.0034	0.0033
rs12678919	a	g	0.1441	0.0419	0.000591	0.008	-0.1554	0.1702	0.0061	0.0057	0.0056
rs7703051	a	c	0.0822	0.0253	0.001143	0.0727	0.002	0.0057	0.0037	0.0035	0.0034
rs838876	a	g	-0.0873	0.0276	0.001546	-0.003	0.0493	-0.0052	0.0042	0.0039	0.0037
rs2954022	a	c	-0.0744	0.0244	0.002338	-0.0546	0.0399	-0.078	0.0036	0.0034	0.0033
rs10282707	t	c	0.0743	0.0252	0.003238	-0.0084	-0.025	0.0092	0.0038	0.0035	0.0034
rs9989419	a	g	0.0821	0.0283	0.003697	0.0277	-0.1473	0.0243	0.0039	0.0036	0.0035
rs492571	t	c	-0.2227	0.0768	0.003718	0.0033	0.0663	-0.0799	0.0097	0.009	0.0088
rs17508045	t	c	0.1298	0.0453	0.004144	0.0488	-0.0085	-0.0083	0.0066	0.0061	0.006

© 2017 American Medical Association. All rights reserved.

rs499974	a	c	0.0873	0.0323	0.006868	0.0012	-0.0263	-0.009	0.0047	0.0044	0.0042
rs10790162	a	g	0.1217	0.0472	0.009889	0.076	-0.0951	0.2305	0.0072	0.0067	0.0065
rs1800961	t	c	0.1636	0.0668	0.01429	-0.0685	-0.127	-0.0017	0.0106	0.0099	0.0094
rs4465830	a	g	0.0763	0.0312	0.01433	-0.009	0.0597	-0.0534	0.0047	0.0044	0.0043
rs1482852	a	g	0.0686	0.0282	0.01488	0.0029	-0.0209	0.013	0.0038	0.0035	0.0035
rs2255141	a	g	-0.0625	0.0276	0.02368	0.0299	0.0337	-0.0209	0.004	0.0037	0.0036
rs6882076	t	c	-0.0572	0.0255	0.02494	-0.0456	-0.0015	-0.0286	0.0038	0.0035	0.0035
rs6544713	t	c	0.0566	0.0263	0.03171	0.0806	-0.003	0.0126	0.0041	0.0038	0.0037
rs4075205	t	c	0.0529	0.0248	0.03277	-0.0119	0.0224	-0.009	0.0038	0.0035	0.0034
rs174532	a	g	0.0603	0.0284	0.03374	0.0353	0.0212	-0.0159	0.0042	0.0039	0.0038
rs261342	c	g	0.0926	0.0451	0.04005	0.0026	-0.1069	-0.0448	0.0065	0.006	0.0059
rs799160	t	c	0.0518	0.0253	0.04062	0.0045	-0.0133	0.0398	0.0039	0.0037	0.0036
rs2412710	a	g	0.2226	0.1112	0.04529	-0.0024	-0.084	0.0988	0.0148	0.0139	0.0132
rs13107325	t	c	-0.1009	0.0516	0.0503	-0.0156	-0.0708	0.0309	0.0083	0.0078	0.0077
rs17789218	t	c	0.0573	0.0297	0.05369	0.0241	-0.0041	0.0061	0.0043	0.004	0.004

© 2017 American Medical Association. All rights reserved.

rs1515110	t	g	0.0482	0.0255	0.05807	0.0063	-0.0323	0.0265	0.0037	0.0035	0.0034
rs7640978	t	c	0.0816	0.0441	0.0643	-0.0392	0.0003	-0.0178	0.0069	0.0064	0.0063
rs7033354	t	c	-0.0481	0.026	0.06449	-0.0189	0.0154	-0.019	0.0038	0.0035	0.0034
rs1035744	t	c	0.0656	0.036	0.06801	0.0069	-0.0055	0.0207	0.0041	0.0038	0.0038
rs1689797	a	c	-0.0473	0.026	0.0686	0.0142	-0.0358	0.0106	0.0039	0.0036	0.0035
rs4939883	t	c	0.0583	0.0322	0.06999	-0.0211	-0.0799	-0.0052	0.0048	0.0045	0.0043
rs10401969	t	c	0.0845	0.0467	0.07061	0.1184	-0.0128	0.121	0.0072	0.0068	0.0065
rs3822072	a	g	-0.0453	0.0252	0.07218	0.0074	-0.0251	0.0182	0.0037	0.0034	0.0034
rs2000999	a	g	0.0555	0.031	0.0738	0.065	0.0023	0.0185	0.0046	0.0043	0.0042
rs6065311	t	c	-0.0399	0.0245	0.1043	-0.0417	-0.0024	-0.0061	0.0036	0.0034	0.0033
rs2980885	a	g	-0.047	0.0294	0.1096	-0.0313	0.0354	-0.0578	0.0045	0.0042	0.0041
rs6859	a	g	0.0407	0.026	0.1171	0.0835	-0.0176	0.0141	0.0039	0.0036	0.0035
rs11660468	t	c	0.0385	0.0249	0.1224	0.0111	0.0391	-0.0008	0.0037	0.0034	0.0034
rs6489818	a	g	0.0484	0.0314	0.124	0.0281	-0.0004	-0.0036	0.0048	0.0045	0.0043
rs10493326	a	g	-0.0437	0.0285	0.1246	0.0213	-0.0013	0.0311	0.0043	0.004	0.0039

© 2017 American Medical Association. All rights reserved.

rs952044	t	c	0.0395	0.026	0.1279	-0.0033	-0.0231	0.0101	0.0039	0.0037	0.0036
rs1367117	a	g	0.0398	0.0264	0.1318	0.1186	-0.0223	0.0252	0.004	0.0037	0.0036
rs1535	a	g	0.0389	0.0259	0.1324	0.0529	0.0391	-0.0463	0.0038	0.0036	0.0035
rs4660293	a	g	-0.0422	0.0284	0.1363	-0.0105	0.0353	-0.0203	0.0043	0.004	0.0039
rs3761445	a	g	0.0468	0.0318	0.1416	0.0081	-0.0158	0.0232	0.0037	0.0035	0.0034
rs4791641	t	c	-0.0358	0.0246	0.1455	-0.0202	-0.0041	0.0028	0.0037	0.0034	0.0033
rs2293889	t	g	0.0356	0.0249	0.1526	0.0147	-0.0312	0.0062	0.0038	0.0035	0.0034
rs6603981	t	c	0.043	0.0304	0.1566	0.034	0.0039	0.0072	0.0044	0.0042	0.004
rs326214	a	g	0.0364	0.026	0.1613	0.0071	-0.0609	0.0235	0.0047	0.0045	0.0042
rs2328223	a	c	-0.0444	0.0327	0.1745	-0.0299	0.0004	0.0066	0.005	0.0046	0.0045
rs7897379	t	c	0.0331	0.0245	0.1775	-0.0104	-0.0194	0.0274	0.0037	0.0034	0.0033
rs2652834	a	g	0.0518	0.0387	0.1812	0.0019	-0.0285	0.0247	0.0046	0.0043	0.0042
rs653178	t	c	-0.0357	0.0268	0.1839	0.0227	0.0263	-0.0099	0.0038	0.0035	0.0034
rs12670798	t	c	-0.0381	0.0288	0.1859	-0.0344	0.0014	-0.0101	0.0043	0.004	0.0039
rs1883025	t	c	-0.0367	0.028	0.1896	-0.0296	-0.0698	-0.0219	0.0044	0.0041	0.004

© 2017 American Medical Association. All rights reserved.

rs38855	a	g	-0.0319	0.0246	0.195	0.001	-0.0146	0.0187	0.0036	0.0034	0.0033
rs1260326	t	c	0.0313	0.0252	0.2138	0.0206	-0.0113	0.1148	0.0037	0.0035	0.0034
rs2290547	a	g	0.0466	0.0377	0.2158	0.0006	-0.0297	0.0096	0.0049	0.0046	0.0044
rs579459	t	c	0.0373	0.0303	0.2182	-0.0665	-0.0145	0.0137	0.0045	0.0042	0.0041
rs2288002	a	g	-0.0302	0.0247	0.2204	-0.0285	-0.0069	-0.0089	0.0038	0.0035	0.0034
rs3780181	a	g	0.0574	0.0475	0.2261	0.0445	0.0038	-0.0069	0.0074	0.0068	0.0066
rs1250229	t	c	0.0336	0.0278	0.2275	-0.0243	0.0034	-0.0089	0.0042	0.0039	0.0038
rs998584	a	c	0.0344	0.0287	0.231	0.0005	-0.026	0.0293	0.004	0.0038	0.0037
rs1010167	c	g	0.034	0.0289	0.2396	-0.025	0.0044	-0.0016	0.0039	0.0037	0.0035
rs3817588	t	c	0.0365	0.0312	0.2418	0.0255	-0.0049	0.0674	0.0046	0.0043	0.0042
rs17173637	t	c	-0.0495	0.0429	0.2487	-0.0069	0.0363	-0.0211	0.0062	0.0057	0.0056
rs4722551	t	c	0.0389	0.0343	0.2565	-0.0391	-0.0101	0.0267	0.0049	0.0045	0.0044
rs2472509	t	g	-0.0298	0.0264	0.2585	-0.0004	-0.0227	0.0024	0.0039	0.0037	0.0036
rs1781930	a	g	-0.0349	0.0316	0.2691	-0.0102	-0.0018	-0.0314	0.0048	0.0045	0.0043
rs17145738	t	c	-0.0421	0.0382	0.2701	0.0039	0.0408	-0.1149	0.0057	0.0053	0.0053

© 2017 American Medical Association. All rights reserved.

rs4976033	a	g	-0.0292	0.0266	0.2723	0.001	0.0215	-0.0141	0.004	0.0037	0.0036
rs4983559	a	g	0.0292	0.0267	0.2732	-0.0026	-0.0197	0.0001	0.0038	0.0036	0.0035
rs7832643	t	g	0.0275	0.0253	0.2765	0.0339	-0.001	0.0017	0.0038	0.0035	0.0034
rs6450176	a	g	0.0303	0.028	0.2794	0.0104	-0.0254	0.0191	0.0042	0.0039	0.0038
rs7607980	t	c	0.0379	0.0366	0.3003	0.0065	-0.0447	0.0362	0.0056	0.0052	0.005
rs267733	a	g	0.0356	0.0344	0.3017	0.0331	-0.0161	0.0025	0.0053	0.005	0.0048
rs4587594	a	g	0.0262	0.0258	0.3098	-0.0493	-0.0147	-0.0694	0.0039	0.0036	0.0035
rs9693857	t	c	-0.0265	0.0266	0.3187	-0.0046	-0.0037	0.0202	0.0037	0.0035	0.0034
rs1169288	a	c	0.0289	0.0294	0.325	-0.0375	-0.0096	-0.0025	0.004	0.0037	0.0036
rs2587534	a	g	-0.0242	0.0247	0.3275	0.0391	0.0093	0.0041	0.0037	0.0034	0.0033
rs4240624	a	g	-0.0442	0.0453	0.3292	0.0672	0.0818	-0.0278	0.0063	0.0058	0.0057
rs12145743	t	g	-0.0254	0.0261	0.3297	0.0042	-0.0203	0.0123	0.0039	0.0036	0.0036
rs17695224	a	g	-0.0267	0.0277	0.3345	-0.0107	-0.029	0.0118	0.0042	0.0039	0.0038
rs10513688	a	g	-0.0394	0.0416	0.3435	0.0222	-0.0049	0.0306	0.0061	0.0057	0.0056
rs2602836	a	g	0.0229	0.0247	0.3544	-0.0007	0.0192	-0.009	0.0037	0.0034	0.0033

© 2017 American Medical Association. All rights reserved.

rs10861661	a	c	-0.0267	0.0288	0.3548	-0.0004	0.022	-0.0227	0.0045	0.0042	0.0041
rs634869	t	c	0.023	0.0249	0.3566	0.0127	-0.0234	0.0272	0.0037	0.0034	0.0033
rs12801636	a	g	0.0267	0.0292	0.3607	0.0078	0.0235	-0.0177	0.0045	0.0042	0.004
rs2737252	a	g	0.0251	0.0275	0.3612	-0.0314	-0.0126	-0.0092	0.0041	0.0038	0.0037
rs17345563	a	g	0.0348	0.0385	0.3665	0.0358	-0.0143	0.015	0.0057	0.0053	0.0052
rs6805251	t	c	0.0224	0.025	0.3707	0.012	0.02	-0.0011	0.0037	0.0035	0.0034
rs2287623	a	g	-0.0222	0.0251	0.3767	-0.0217	-0.0112	0.0006	0.0037	0.0035	0.0034
rs2923084	a	g	-0.028	0.0317	0.3772	-0.0121	0.0256	-0.0119	0.0049	0.0045	0.0044
rs13326165	a	g	-0.03	0.0341	0.3788	-0.0042	0.0289	-0.0205	0.0046	0.0043	0.0042
rs4530754	a	g	-0.0215	0.0245	0.3802	0.0275	0.0008	0.0015	0.0036	0.0034	0.0033
rs8176720	t	c	0.0226	0.0259	0.384	0.0332	0.0005	-0.0073	0.0038	0.0036	0.0035
rs3996352	a	g	-0.0212	0.0245	0.3874	0.0053	-0.0296	0.0179	0.0037	0.0034	0.0033
rs5880	c	g	0.0484	0.0575	0.4002	0.047	-0.3071	0.0475	0.0096	0.009	0.0085
rs11045163	a	g	-0.0204	0.0252	0.4178	0.0055	-0.0217	0.0097	0.0037	0.0035	0.0034
rs8077889	a	c	-0.0244	0.0303	0.4207	-0.0005	0.0205	-0.0252	0.0046	0.0043	0.0042

© 2017 American Medical Association. All rights reserved.

rs868943	a	g	-0.0201	0.025	0.4208	-0.0264	-0.0075	-0.0137	0.0037	0.0035	0.0034
rs4846914	a	g	-0.0201	0.0255	0.4287	-0.0043	0.0479	-0.0401	0.0037	0.0034	0.0034
rs492602	a	g	-0.0201	0.0257	0.4334	-0.0293	0.0032	-0.0139	0.0039	0.0036	0.0035
rs1186380	t	c	0.0243	0.0313	0.4389	-0.0244	-0.0002	0.0026	0.0043	0.004	0.0039
rs7264396	t	c	-0.0231	0.0301	0.4426	-0.0246	-0.0054	-0.0108	0.0045	0.0042	0.0041
rs2278236	a	g	-0.0183	0.0248	0.4595	0.0067	0.0331	-0.0141	0.0038	0.0035	0.0034
rs10019888	a	g	0.0245	0.0338	0.4681	-0.0182	0.027	-0.0228	0.005	0.0046	0.0045
rs2240327	a	g	0.0176	0.0243	0.4708	-0.0005	-0.0242	0.0017	0.0036	0.0034	0.0033
rs12525163	t	c	-0.0196	0.0271	0.4711	0.0043	-0.0215	0.0086	0.004	0.0037	0.0037
rs217386	a	g	0.0167	0.0248	0.5003	-0.0363	0.0013	-0.0101	0.0038	0.0035	0.0034
rs2326077	t	c	-0.0173	0.026	0.5043	-0.0341	-0.0043	-0.018	0.0038	0.0035	0.0034
rs2030746	t	c	-0.0167	0.0251	0.5055	0.0214	-0.0025	0.0031	0.0038	0.0036	0.0035
rs1688030	t	c	0.0322	0.0487	0.5083	-0.0163	-0.0085	-0.0378	0.0076	0.007	0.0068
rs749671	a	g	-0.0165	0.0255	0.5171	-0.0152	0.0071	-0.0211	0.0037	0.0035	0.0034
rs6016381	t	c	0.0163	0.0253	0.5186	0.0363	-0.0084	0.0144	0.0038	0.0035	0.0034

© 2017 American Medical Association. All rights reserved.

rs9930333	t	g	-0.0159	0.0247	0.5199	0.0002	0.0199	-0.0208	0.0041	0.0037	0.0037
rs2710642	a	g	-0.0163	0.0261	0.5314	0.0239	-0.0096	0.0065	0.0038	0.0035	0.0034
rs2297374	t	c	-0.0155	0.0254	0.5405	-0.0325	0.0056	-0.0091	0.0038	0.0035	0.0034
rs686030	a	c	0.0213	0.0353	0.5467	0.0085	0.055	0.025	0.0052	0.0049	0.0048
rs688	t	c	0.0141	0.0245	0.5633	0.054	-0.0106	0.0041	0.0037	0.0034	0.0034
rs4148005	t	g	0.0149	0.0263	0.5711	-0.015	0.0283	-0.0066	0.0039	0.0036	0.0035
rs9875338	a	g	0.0142	0.025	0.5715	-0.027	-0.0073	-0.0144	0.0037	0.0035	0.0034
rs6680658	a	g	-0.0164	0.029	0.5717	-0.0055	0.0232	-0.0166	0.0045	0.0041	0.004
rs17286602	a	t	-0.0135	0.0246	0.5821	-0.0032	0.021	-0.006	0.0037	0.0034	0.0033
rs4921914	t	c	0.0161	0.0296	0.5856	-0.0229	-0.0019	-0.0353	0.0044	0.0041	0.004
rs16942887	a	g	0.0202	0.0375	0.5905	0.0011	0.0831	-0.0115	0.0054	0.0051	0.005
rs2294261	a	c	0.0142	0.0265	0.5916	0.0333	-0.0085	0.0021	0.0037	0.0035	0.0034
rs2073547	a	g	-0.0169	0.0321	0.5983	-0.0485	0.0049	-0.0148	0.0049	0.0045	0.0044
rs7117842	t	c	0.0131	0.0253	0.6045	-0.0192	-0.0272	0.002	0.0038	0.0035	0.0034
rs10903129	a	g	0.0123	0.0246	0.6163	-0.0328	-0.0009	-0.008	0.0037	0.0034	0.0033

© 2017 American Medical Association. All rights reserved.

rs4650994	a	g	-0.0121	0.0243	0.6199	0.0027	-0.021	0.0024	0.0036	0.0034	0.0033
rs2925979	t	c	0.013	0.0265	0.6243	-0.0031	-0.0351	0.0205	0.004	0.0037	0.0036
rs364585	a	g	0.0122	0.025	0.6263	-0.0249	-0.0005	0.0018	0.0038	0.0035	0.0034
rs3741414	t	c	-0.014	0.0288	0.6268	-0.0159	0.0296	-0.028	0.0043	0.004	0.0039
rs7225700	t	c	0.0125	0.0257	0.6277	-0.0297	-0.0098	0.0046	0.0038	0.0035	0.0035
rs1800562	a	g	-0.0227	0.0493	0.6444	-0.0615	-0.0074	0.013	0.008	0.0074	0.0072
rs731839	a	g	0.0121	0.0263	0.6461	0.0018	0.022	-0.0224	0.004	0.0037	0.0036
rs12133576	a	g	0.0099	0.0254	0.6975	0.0104	0.0243	-0.009	0.0038	0.0035	0.0034
rs2303975	a	g	-0.0141	0.0378	0.7087	-0.0013	0.0279	-0.0115	0.0053	0.0049	0.0048
rs702485	a	g	-0.0088	0.0257	0.7319	-0.001	-0.0243	0.0016	0.0037	0.0034	0.0033
rs6831256	a	g	0.0078	0.0236	0.7402	-0.0188	0.0131	-0.0258	0.0038	0.0036	0.0035
rs103294	t	c	0.0098	0.0314	0.7557	0.0073	0.0523	-0.0021	0.0047	0.0044	0.0042
rs442177	t	g	-0.0077	0.025	0.7565	0.0159	-0.0215	0.0309	0.0037	0.0034	0.0033
rs9686661	t	c	0.0096	0.0314	0.7602	0.0179	-0.0283	0.0379	0.0048	0.0044	0.0044
rs10102164	a	g	0.0092	0.0302	0.7603	0.0316	-0.0005	0.0113	0.0045	0.0042	0.0041

© 2017 American Medical Association. All rights reserved.

rs16831243	t	c	-0.0114	0.0387	0.7687	0.0378	0.0114	-0.0006	0.0055	0.0051	0.0051
rs355838	t	g	0.0064	0.0252	0.7998	0.0178	-0.0191	0.0135	0.0038	0.0035	0.0034
rs4942486	t	c	-0.0059	0.0245	0.8106	0.0243	-0.0136	0.0071	0.0037	0.0034	0.0033
rs9491696	c	g	0.0058	0.0244	0.8132	-0.0057	0.0203	-0.0179	0.0037	0.0034	0.0034
rs2642438	a	g	-0.0064	0.0277	0.8162	-0.0352	-0.0303	0.0172	0.0042	0.0039	0.0038
rs2068888	a	g	-0.0057	0.025	0.8194	-0.0166	0.0193	-0.0241	0.0038	0.0035	0.0034
rs1532085	a	g	0.0071	0.032	0.8249	0.0026	0.1068	0.031	0.0037	0.0035	0.0034
rs1341267	a	c	0.0051	0.0248	0.8383	0.0016	0.0023	-0.0184	0.0037	0.0034	0.0033
rs11246602	t	c	-0.0078	0.0392	0.842	-0.0019	-0.034	0.0091	0.0056	0.0052	0.0052
rs10773105	t	c	0.0049	0.025	0.8446	0.0058	-0.0363	0.0037	0.0037	0.0035	0.0034
rs11220462	a	g	0.007	0.0358	0.8458	0.059	-0.0162	0.0189	0.0059	0.0056	0.0054
rs687339	t	c	-0.0055	0.0292	0.8513	0.011	-0.0316	0.0291	0.0045	0.0042	0.004
rs8017377	a	g	0.0043	0.0252	0.8644	0.0303	-0.0037	0.0056	0.0038	0.0036	0.0035
rs10832962	t	c	-0.0047	0.0278	0.8662	0.032	0.0043	0.0109	0.004	0.0038	0.0037
rs10029254	t	c	0.0048	0.0286	0.8666	0.0058	-0.0085	0.0266	0.0044	0.0041	0.0039

© 2017 American Medical Association. All rights reserved.

rs903319	t	c	0.0044	0.0275	0.8725	-0.0266	-0.0103	0.0054	0.0041	0.0038	0.0037
rs314253	t	c	0.0041	0.0258	0.8751	0.0242	-0.003	0.0086	0.0038	0.0035	0.0034
rs970548	a	c	-0.0042	0.0283	0.8822	-0.0157	-0.0258	-0.0025	0.0042	0.0039	0.0038
rs4148218	a	g	-0.0045	0.0317	0.886	-0.0441	0.0029	-0.0037	0.0047	0.0044	0.0042
rs4969178	a	g	-0.0034	0.0253	0.8944	-0.011	-0.0263	0.0175	0.0037	0.0035	0.0034
rs7254892	a	g	-0.0104	0.083	0.9	-0.4853	0.0528	0.1235	0.0119	0.0113	0.0106
rs5763662	t	c	0.0127	0.1093	0.9078	0.0767	0.0333	-0.0001	0.0121	0.0112	0.0112
rs1998013	t	c	0.0235	0.2154	0.9131	-0.3806	0.0354	0.0089	0.022	0.0201	0.0199
rs3198697	t	c	-0.0025	0.0248	0.9204	0.0096	0.0163	-0.0198	0.0037	0.0035	0.0034
rs17788930	a	g	-0.0024	0.0258	0.925	0.0046	0.0359	-0.0112	0.0038	0.0036	0.0035
rs4871137	t	g	-0.0024	0.0262	0.9262	-0.0043	-0.0209	-0.0013	0.0039	0.0037	0.0036
rs4917014	t	g	0.0014	0.0262	0.956	-0.0047	-0.0222	0.0012	0.0039	0.0036	0.0035
rs11563251	t	c	0.0021	0.0402	0.9589	0.0345	0.0058	0.0083	0.0062	0.0057	0.0056
rs2241210	a	g	-0.0012	0.0245	0.9617	-0.0078	-0.0332	-0.0029	0.0037	0.0035	0.0033
rs205262	a	g	-0.001	0.0282	0.9713	0.0088	0.0283	-0.0028	0.0041	0.0039	0.0038
rs122	a	g	-0.001	0.035	0.975	-0.000	-0.033	0.006	0.005	0.004	0.005

© 2017 American Medical Association. All rights reserved.

26802			1	9	2	2	1	7	3	9	
rs1564348	t	c	-0.001	0.0327	0.9765	-0.0481	0.0077	-0.0164	0.005	0.0046	0.0045
rs603446	t	*c*	-0.0004	0.0246	0.9874	-0.0092	0.0018	-0.0502	0.0037	0.0035	0.0034

© 2017 American Medical Association. All rights reserved.

eTable 2. Association of Lipid GRS with CHD in the CARDIoGRAMplusC4D Dataset Using Inverse Weighted MR and MR-Egger

Lipid trait	# of SNPS	Inverse variance weighted OR (lower, upper 95%CI)	MR-Egger OR (lower, upper 95%CI)	MR-Egger intercept P-value
LDL-cholesterol	75	1.63 (1.45, 1.83)	1.83 (1.49, 2.25)	0.16
HDL-cholesterol	84	0.76 (0.66,0.87)	0.96 (0.75, 1.22)	0.02
Triglycerides	50	1.51 (1.29, 1.76)	1.46 (1.12, 1.89)	0.75

© 2017 American Medical Association. All rights reserved.

eReferences

1. Jones GT, Tromp G, Kuivaniemi H et al. Meta-Analysis of Genome-Wide Association Studies for Abdominal Aortic Aneurysm Identifies Four New Disease-Specific Risk Loci. Circulation research 2017;120:341-353.
2. Bown MJ, Jones GT, Harrison SC et al. Abdominal aortic aneurysm is associated with a variant in low-density lipoprotein receptor-related protein 1. Am J Hum Genet 2011;89:619-27.
3. Borthwick KM, Smelser DT, Bock JA et al. ePhenotyping for Abdominal Aortic Aneurysm in the Electronic Medical Records and Genomics (eMERGE) Network: Algorithm Development and Konstanz Information Miner Workflow. Int J Biomed Data Min 2015;4.
4. Verma SS, de Andrade M, Tromp G et al. Imputation and quality control steps for combining multiple genome-wide datasets. Front Genet 2014;5:370.
5. Delaneau O, Marchini J, Zagury JF. A linear complexity phasing method for thousands of genomes. Nat Methods 2012;9:179-81.
6. Howie B, Fuchsberger C, Stephens M, Marchini J, Abecasis GR. Fast and accurate genotype imputation in genome-wide association studies through pre-phasing. Nat Genet 2012;44:955-9.
7. Purcell S, Neale B, Todd-Brown K et al. PLINK: a tool set for whole-genome association and population-based linkage analyses. American journal of human genetics 2007;81:559-75.
8. Gretarsdottir S, Baas AF, Thorleifsson G et al. Genome-wide association study identifies a sequence variant within the DAB2IP gene conferring susceptibility to abdominal aortic aneurysm. Nature Genetics 2010;42:692-U71.
9. Gretarsdottir S, Thorleifsson G, Reynisdottir ST et al. The gene encoding phosphodiesterase 4D confers risk of ischemic stroke. Nature genetics 2003;35:131-8.
10. JA Rice. Generalized likelihood ratio tests. Mathematical Statistics and Data Analysis Vol 1 International Thomson Publishing 1995:308-310.
11. Howie B, Marchini J, Stephens M. Genotype imputation with thousands of genomes. G3 (Bethesda) 2011;1:457-70.
12. Devlin B, Roeder K. Genomic control for association studies. Biometrics 1999;55:997-1004.
13. Simons PC, Algra A, van de Laak MF, Grobbee DE, van der Graaf Y. Second manifestations of ARTerial disease (SMART) study: rationale and design. Eur J Epidemiol 1999;15:773-81.
14. Schunkert H, Konig IR, Kathiresan S et al. Large-scale association analysis identifies 13 new susceptibility loci for coronary artery disease. Nature genetics 2011;43:333-8.

© 2017 American Medical Association. All rights reserved.
